# Direct Precursor Route for the Fabrication of LLZO Composite Cathodes for Solid‐State Batteries

**DOI:** 10.1002/advs.202404682

**Published:** 2024-09-19

**Authors:** Vivien Kiyek, Christian Schwab, Walter Sebastian Scheld, Christoph Roitzheim, Adrian Lindner, Wolfgang Menesklou, Martin Finsterbusch, Dina Fattakhova‐Rohlfing, Olivier Guillon

**Affiliations:** ^1^ Institute of Energy Materials and Devices—Materials Synthesis and Processing (IMD‐2) Forschungszentrum Jülich GmBH 52425 Jülich Germany; ^2^ Institute of Mineral Engineering RWTH Aachen University 52064 Aachen Germany; ^3^ Institute for Applied Materials—Electrochemical Technologies (IAM‐ET) Karlsruhe Institute of Technology (KIT) 76131 Karlsruhe Germany; ^4^ Helmholtz Institute Münster—Ionics in Energy Storage (IEK‐12) 48149 Münster Germany; ^5^ Faculty of Engineering and Center for Nanointegration Duisburg‐Essen University Duisburg‐Essen 47057 Duisburg Germany; ^6^ Jülich‐Aachen Research Alliance: JARA‐ENERGY 52425 Jülich Germany

**Keywords:** all‐solid–state batteries, ceramic composites, in situ synthesis, LLZO

## Abstract

Solid–state batteries based on Li_7_La_3_Zr_2_O_12_ (LLZO) garnet electrolyte are a robust and safe alternative to conventional lithium‐ion batteries. However, the large‐scale implementation of ceramic composite cathodes is still challenging due to a complex multistep manufacturing process. A new one‐step route for the direct synthesis of LLZO during the manufacturing of LLZO/LiCoO_2_ (LCO) composite cathodes based on cheap precursors and utilizing the industrially established tape casting process is presented. It is shown that Al, Ta:LLZO can be formed directly in the presence of LCO from metal oxide precursors (LiOH, La_2_O_3_, ZrO_2_, Al_2_O_3_, and Ta_2_O_5_) by heating to 1050 °C, eliminating the time‐ and energy‐consuming synthesis of preformed LLZO powders. In addition, performance‐optimized gradient microstructures can be produced by sequential casting of slurries with different compositions, resulting in dense and flat phase‐pure cathodes without unwanted ion interdiffusion or secondary phase formation. Freestanding cathodes with a thickness of 85 µm, a relative density of 95%, and an industrial relevant LCO loading of 15 mg show an initial capacity of 82 mAh g^−1^ (63% of the theoretical capacity of LCO) in a solid‐state cell with Li metal anodes, which is comparable to conventional LCO/LLZO cathodes and can be further improved in the future.

## Graphical abstract

Free‐standing solid‐state cathodes of Li_7_La_3_Zr_2_O_12_ (LLZO) solid electrolyte and LiCoO_2_ (LCO) are formed in a one‐step process based on cheap precursors and utilizing the industrially established tape casting process. LLZO can be formed directly in the presence of LCO from metal oxide precursors by heating to 1050 °C, eliminating the time‐ and energy‐consuming synthesis of preformed LLZO powders.

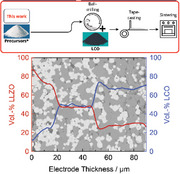

## 1 Introduction

Solid–state batteries with the garnet electrolyte Li_7_La_3_Zr_2_O_12_ (LLZO) are attracting a lot of attention as a robust and safe alternative to conventional lithium‐ion batteries. As a non‐combustible ceramic material with excellent thermal, chemical, and electrochemical stability, LLZO offers the highest level of safety at cell level and can potentially be used to develop batteries for applications in harsh conditions, including high‐temperature applications.^[^ ^1^ ^]^ One of the key properties of LLZO is its high stability to Li‐metal, making it a very attractive separator and anolyte material for high energy cells with Li‐metal anodes.^[^ ^2^ ^]^ The excellent performance of LLZO has recently been demonstrated in cells with Li‐metal electrodes, where stable cycling with critical current densities of up to 100 mA cm^−2^ has been achieved.^[^ ^3^ ^]^

LLZO is not only successfully applied as a separator in anode half cells, but also exhibits sufficient ionic conductivity to be used as a catholyte in fully inorganic batteries without containing flammable organic components, thus achieving the highest possible safety at cell level. To achieve the required high loading of active cathode material (CAM), composite electrode morphologies with an interconnected network of LLZO and CAM are required, providing ionic and electronic conductivity pathways, respectively, to enable rapid lithiation/de‐lithiation of even thick cathodes.^[^ ^4^, ^5^ ^]^

The challenges in the practical realization of oxide solid‐state batteries in case of composite cathodes are related to the fact that LLZO and CAM are rigid ceramic materials that require a special design to obtain the desired electrode morphology. In contrast to cells with liquid electrolytes, which can easily conform to the desired shape and spontaneously wet the CAM to create a conductive interface, LLZO must be sintered at high temperatures to establish contact with the CAM with a sufficiently low interfacial impedance.^[^ ^6^ ^]^ Since the sintering process is usually driven by diffusion, it can also trigger a number of undesirable processes. It has long been known that the thermal stability of CAMs decreases significantly in the presence of LLZO with the formation of various reaction products.^[^ ^6^, ^7^, ^8^, ^9^, ^10^, ^11^ ^]^ Among the numerous CAMs, LiCoO_2_ (LCO) exhibits the highest thermal stability with LLZO and is so far the only known material that can be successfully sintered in combination with LLZO powders to form ceramic composite cathodes with good performance and without any sintering aids.^[^ ^6^, ^12^, ^13^, ^14^, ^15^ ^]^ However, numerous material interactions can also occur in the LCO‐LLZO system during sintering, leading to material degradation during processing and affecting the cell performance. One of these processes is the interdiffusion of highly mobile ions such as Al (from Al‐doped LLZO) and Co (from LCO) with the formation of substituted or doped phases.^[^ ^7^, ^9^, ^16^, ^17^ ^]^ In addition, reactions with the formation of various secondary phases such as Li_0.5_La_2_Co_0.5_O_4_ or Co‐doped LLZO are frequently observed, leading to an additional loss of capacity.

Due to their great importance for the performance of oxide solid‐state batteries, the development and production of ceramic cathodes has attracted a great deal of attention. To date, numerous publications have appeared on the processing, sintering, and shaping of ceramic cathodes.^[^ ^18^ ^]^ The approaches to producing the cathode layers in LLZO‐based cells can be divided into three main groups. In the first group, the CAM is deposited as a thin film on a dense ceramic LLZO separator by physical vapor deposition or wet chemical deposition.^[^ ^13^, ^19^, ^20^, ^21^, ^22^, ^23^, ^24^ ^]^ However, efficient charge transport in dense electrodes is limited to very low thicknesses and thus low areal capacities of the CAM, which makes them impractical for applications requiring high energy density.

In the second group, LLZO and CAM powders are mixed and sintered together after the cathodes have been formed using various techniques (from cathode layers deposited on ceramic separators to self‐supporting cathodes). This approach is the most popular as it is practical, offers a wide range of possibilities to vary the cathode morphology and a variety of sintering techniques can be used to densify the electrodes. One of the major drawbacks of the co‐sintering strategy is the vastly different temperatures required to densify LLZO and CAM powders, as well as undesirable material interactions such as ion interdiffusion and secondary phase formation as described above. Advances in sintering techniques have led to a large number of different LLZO‐based cathode morphologies in recent years, where processing‐induced material degradation has been minimized, resulting in ceramic cathodes with sufficiently good performance. Reported processes include the deposition of cathode inks by ink or screen printing on LLZO separators^[^ ^15^, ^25^ ^]^ or pressure‐assisted sintering of cathode half‐cells by field‐assisted sintering/spark plasma sintering^[^ ^26^ ^]^ as well as a wide range of sintering techniques ranging from conventional sintering^[^ ^27^, ^28^ ^]^ to ultrafast^[^ ^29^ ^]^ and photonic sintering.^[^ ^25^ ^]^

Finally, the last group of techniques is based on the in situ formation of CAM in the pores of a preformed porous matrix of LLZO. Due to the much lower temperatures required for CAM formation (≈600 °C), this approach allows minimizing unwanted reactions and provides flexibility in designing the cathode morphology by tuning the porosity of the LLZO framework.^[^ ^30^ ^]^

All approaches described so far for the preparation of LLZO‐based composite cathodes are based on the use of crystalline LLZO powders with defined chemical and phase composition and high ionic conductivity, which can be prepared by various methods such as solid‐state reaction, sol–gel process, spray pyrolysis, or coprecipitation.^[^ ^1^, ^31^, ^32^, ^33^, ^34^, ^35^, ^36^ ^]^ The use of preformed LLZO powder is rationalized by the synthesis conditions, which require high temperatures of at least 500 °C (usually above 1000 °C) and long heating times in several calcination and sintering steps to obtain phase‐pure cubic LLZO with high ionic conductivity.^[^ ^37^ ^]^ In addition to the problems related to the stability of CAMs, the synthesis of LLZO is very time and energy demanding, resulting in high energy consumption, high cost, and high environmental impact of the entire LLZO‐based battery manufacturing process. Therefore, simplifying the manufacturing process with a complete reduction in processing time and energy savings is very important for the successful development of oxide solid‐state batteries, in this case for composite cathodes.

Contrary to a widely held belief about the need to use phase‐pure LLZO material for cathode fabrication, we show that LLZO can be formed directly during cathode processing in the presence of CAM. We present a new precursor route based on cheap, non‐toxic oxide precursors for the direct (in situ) synthesis of LLZO during sintering of a composite cathode. We show that this approach can be used in an industrially relevant tape casting process that enables the continuous production of free‐standing ceramic cathodes on a large scale. The direct use of the LLZO precursors (LiOH, La_2_O_3_, ZrO_2_, Al_2_O_3_, and Ta_2_O_5_) in the tape casting process enables the LLZO synthesis and the preparation of the composite cathode in a single step. The in situ approach described in this work leads to dense and flat freestanding cathode tapes with a strong suppression of unwanted ion interdiffusion or secondary phase formation. Compared to the known processes for the composite cathode fabrication described above, only one heat treatment step is required in a direct precursor synthesis of composite cathodes, saving energy and time. The method eliminates the time‐consuming synthesis of pre‐formed LLZO powders for the subsequently production of a composite cathode. The presented method is very effective in producing high‐quality LLZO in dense composite cathode tapes and achieves comparable performance to conventional methods. The new precursor route is therefore faster, simpler, and more energy efficient, which can contribute to the development of sustainable and cost‐effective solid‐state batteries based on ceramic materials.

## 2 Results and Discussion

### 2.1 Fabrication and Characterization of LCO‐LLZO Composite Cathode Tapes

**Figure 1:**
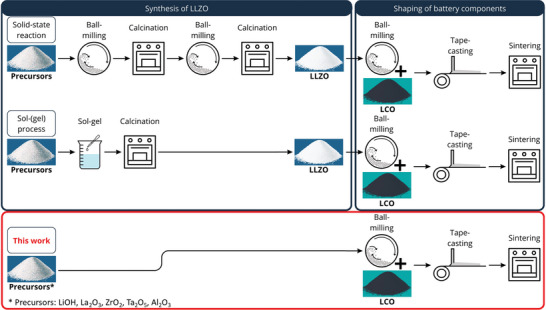
Scheme of solid‐state reaction, sol–gel process, and the route shown in this work followed by a tape‐casting and sintering step.

In order to compare conventional methods for the preparation of ceramic cathodes with the newly developed ones, the workflows for the established industrial tape casting process are shown in **Figure** **1**. Established processes for the fabrication of composite ceramic LCO‐LLZO cathodes use pre‐synthesized LCO and LLZO powders with defined crystallinity and phase composition as CAM and solid electrolyte (SE), respectively.^[^ ^13^, ^15^, ^16^, ^17^, ^19^, ^23^, ^38^ ^]^ The LLZO powder is usually synthesized by a solid‐state or a sol–gel reaction in a separate procedure that requires several milling and heating steps to obtain LLZO with sufficient phase purity and ionic conductivity.

In contrast to the established methods, the route described here does not use pre‐synthesized LLZO powder. Instead, the starting materials for LLZO synthesis (pre‐LLZO) are added directly to the slurry together with the LCO powder, and the formation of LLZO is expected to take place in situ during heating steps. The stoichiometry and chemical composition of the desired LLZO phase can be adjusted by the choice of starting materials and their concentration. To produce Al‐ and Ta‐doped LLZO (LLZO:Ta,Al), which is known to have sufficiently high ionic conductivity and electrochemical stability, LiOH, La_2_O_3_, ZrO_2_, Al_2_O_3_, and Ta_2_O_5_ were added to the slurry as LLZO precursors. The relative ratios of starting materials were adjusted to ensure the formation of Li_6.54_Al_0.02_La_3_Zr_1.6_Ta_0.4_O_12_ as the target composition. Figure 1 shows a comparison between a solid‐state reaction and a sol–gel process for the synthesis of LLZO powder followed by tape‐casting and the precursor route presented in this work to illustrate the idea behind the approach and to show the minimization of processing steps.

In a typical process, a slurry containing the ceramic powders and a mixture of organic binders, plasticizers, and dispersants is cast onto a Mylar foil to produce 10 to 100 µm thick tapes after drying. The flexible “green tapes,” which can be detached from the carrier foil, must undergo heat treatment to burn out the organic components (usually in a separate debinding step at temperatures around 600 °C) and to compact the ceramic powder at higher temperature in a sintering step to produce fully ceramic, free‐standing cathode tapes. In addition to the relative proportions of LLZO precursors, their concentration in the slurry can be varied to achieve different ratios of LLZO and LCO phases in the resulting composite cathodes, which strongly affects their charge transport properties (ionic and electronic conductivity).^[^ ^39^, ^40^ ^]^ Besides composite cathode morphologies containing a homogeneous distribution of LCO and LLZO phases, gradient structures can be prepared by sequential casting of slurries with different compositions.^[^ ^28^ ^]^

To produce the gradient cathode tapes described in this work, we sequentially cast three slurry compositions containing a precursor mixture for Al, LLZO:Ta, and LCO in different ratios to obtain layers with LLZO:LCO ratios of 2:1, 1:1, and 1:2 after sintering (an average LLZO:LCO ratio in the tape was set at 50:50 wt%). The tape casting process was adjusted to produce a green tape with a thickness of 100 µm. The green tape was sintered in an alumina crucible on a mixture of sacrificial LLZO and LCO powders with two holding steps at 200 and 650 °C for 1 and 2 h, respectively, and a final sintering at 1050 °C for 2 h, resulting in fully ceramic mechanically stable tape with a thickness of 85 µm. A temperature profile of the heat treatment is shown in Figure S1 (Supporting information).

**Figure 2:**
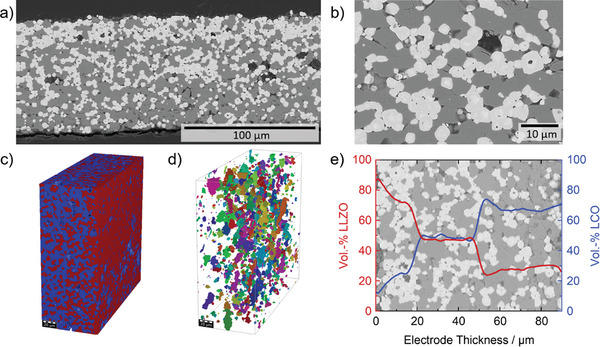
a) BSE‐SEM image over a cross‐section with the full thickness revealing the gradient structure of the composite cathode with a high LLZO area and a high LCO amount area. b) BSE‐SEM image over a cross‐section with higher resolution, showing the interfaces between LLZO and LCO particles. Results of micro X‐ray tomography (µCT) analysis of the layered electrode in c) Segmented 3D structure with a visible gradient from LLZO (red) rich side to LCO (blue) rich side. d) Analysis of pore connectivity performed with GeoDict. Different colors represent unconnected pore clusters. e) Distribution of solid electrolyte (LLZO) and active material (LCO) along the electrode thickness quantified via GeoDict. The layered structure is visible and both LLZO‐rich and LCO‐rich as well as the mixed layer are shown, while the BSE‐SEM image from a cross‐section is in the background.

Microstructural analysis of the sintered tapes using backscattered electron scanning electron microscopy (BSE‐SEM) shows that the chosen process indeed leads to the formation of dense tapes with a gradient distribution of two phases, referred to as LCO (dark contrast) and LLZO (bright contrast) (**Figure** **2**a). Both phases consist of interconnected particles that are well sintered together and provide intertwined percolation pathways for Li transport. Higher resolution BSE‐SEM images show that sintering necks with sharp and defined interfaces also form between the particles of the different phases (Figure 2b). The LLZO phase consists of relatively large particles with a size of 5–6 µm (D_50_), similar to the LCO particle size (Figure S2, Supporting Information), which is larger than the particle size of the milled LLZO precursor particles added to the slurry. BSE‐SEM images show oxide particles with a primary particle size of <1 µm (Figure S3, Supporting Information). LiOH is detected as a hydrated phase as shown by the markers in the figure.

Micro X‐ray tomography (µCT) analysis confirms the formation of a segmented 3D electrode morphology with a visible compositional gradient after sintering (Figure 2c–e). The ratio of LCO:LLZO is ≈2:1, followed by a ratio of 1:1 as targeted in the fabrication process. The LLZO‐rich side shows a gradient toward the edge, with increasing LLZO and decreasing LCO amount (Figure 2e). The relative density of the composite cathode tapes is 95%, which is high for the tapes sintered in a free process without pressure assistance and higher than the average density of cathode tapes made of pre‐formed LLZO particles, which is generally below 95%. The analysis of porosity in µCT shows that the pores are not interconnected (closed porosity), although different diameters of either small pores with <1 µm or large pores in the range of 10 µm are present (Figure 2d). Due to the lower melting temperature and higher sintering activity of the LCO phase, it can be assumed that the LCO particles sinter earlier than the LLZO particles and form a 3D LCO matrix, which serves as a host for the formation of LLZO domains. Due to the rigid structure of the LCO submatrix and the limited diffusion pathways, not all of the available space is filled with LLZO, resulting in large pores where the material is absent. Pores or delamination are not observed between layers with different compositions, indicating good wetting of already cast layers with a slurry in a sequential tape casting process (Figure 2e).

**Figure 3:**
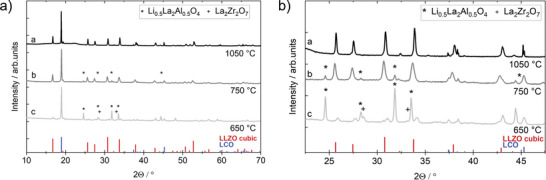
X‐ray diffraction (XRD) 2Θ scan of tapes after heat treatment of 650, 750, and 1050 °C with indication of side phases such as Li_0.5_La_2_Al_0.5_O_4_ and La_2_Zr_2_O_7_ and the cubic LLZO structure (ICSD: 182 312) and LCO (ICSD:29 225) as references.

X‐ray diffraction (XRD) analysis of the composite cathode tapes sintered at 1050 °C shows the presence of only LCO and cubic LLZO as two main phases (**Figure** **3**). No precursors of LLZO or secondary phases were detected, confirming that the cubic LLZO phase was successfully formed in situ from metal oxide particles during sintering of the green tapes and that LCO remained stable during LLZO formation. Rietveld refinement of the XRD data from the LLZO‐rich side of the sintered tapes shows that 66% of LLZO:Ta and 34% of LCO are present, which corresponds exactly to the expected 2:1 ratio (Figure S3, Supporting Information).

**Figure 4:**
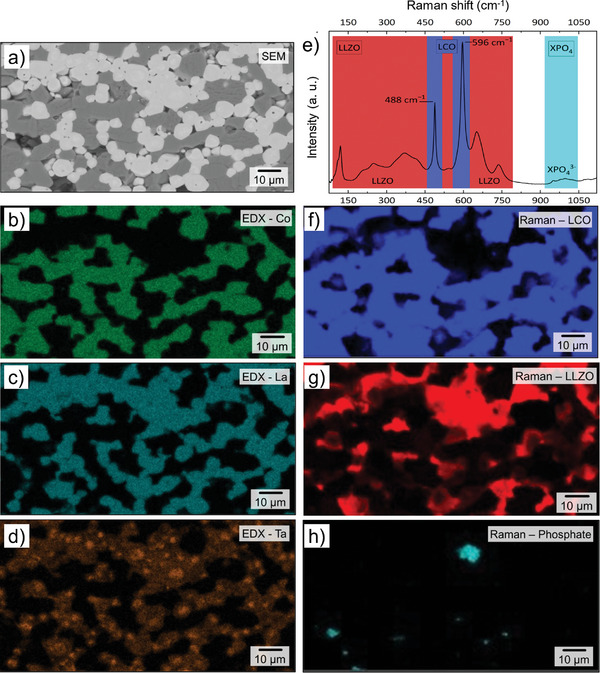
Scanning electron microscopy (a–d) and Raman spectroscopy analysis (e–h) of a LLZO‐rich side of an LCO‐LLZO cathode tape with gradient morphology after sintering at 1050 °C: BSE‐SEM image of the analyzed area (a) and the corresponding elemental distribution of b) Co, c) La, and d) Ta. Raman spectra from the same tape region as in (a): e) averaged Raman spectra with LLZO in red, LCO in blue, and a secondary phase with phosphate (XPO_4_) in turquoise, and the corresponding Raman signal distribution of f) LCO, g) LLZO, and h) the secondary phosphate phase (XPO_4_).

The conclusion of the XRD analysis is largely supported by the BSE‐SEM images (**Figure** **4**a) with Energy Dispersive X‐Ray Analysis (EDS) mapping (Figure 4b–d) and Raman spectroscopy, which demonstrate a high phase purity of the composite cathodes after sintering, although small amounts of impurities can be detected due to higher resolution of these methods. The EDS mapping (Figure 4b–d) of the sintered cathode tapes shows that La is located in the regions of bright contrast assigned to LLZO, and Co is confined to the regions of dark contrast corresponding to LCO, which is consistent with the targeted chemical composition. The sharp contrast between the two phases and the lack of detectable element interdiffusion between the two phases or elemental enrichment/depletion at the interface indicate that the LLZO phase formed adjacent to the LCO phase, with no traces of ionic interdiffusion between the phases or secondary phase formation. The time‐of‐flight secondary ion mass spectrometry (ToF‐SIMS) measurements discussed later also lead to similar results.

Although the SEM analysis confirms that the sintered cathode tapes consist of LCO and LLZO as dominate phases, it also shows the presence of some isolated domains with different contrast that correlate with inhomogeneities in the distribution of Ta ions. Thus, areas of brighter contrast enriched in Ta atoms can be seen in the center of individual LLZO grains. EDS analysis shows that these domains contain mainly Ta and O atoms and practically no La and Co atoms. Raman spectroscopy analyses of these regions do not show signals of phases other than LLZO and LCO (Figure 4e). Since the XRD analysis also does not show secondary phases, the reason for the Ta‐enriched regions could be a slight deviation in the stoichiometry or a partially insufficient initial mixture of precursors. It should be noted that the inhomogeneous Ta distribution, where the LLZO grains show a higher Ta content in the center of the particle and a decreasing Ta gradient toward the particle edge, is common for LLZO:Ta and has been observed for materials synthesized by different methods,^[^ ^17^, ^25^, ^27^, ^34^, ^37^, ^41^ ^]^ and is not unique to the newly developed synthesis route shown here, although this inhomogeneity does not appear to significantly affect LLZO performance. It can be speculated that tantalum oxide particles (or Ta‐rich compounds) act as nucleation centers for the formation and crystallization of the cubic LLZO phase, although the mechanism of this process is still unknown and deserved more detailed investigation in the future.

In addition to Ta inhomogeneities, a small amount of phosphorus was found in the pores using EDS analysis (Figure S4, Supporting Information). Similar results were also obtained in Raman spectroscopy (Figure 4h), which shows signals at 950 and 995 cm^−1^ assigned to symmetric and asymmetric stretching of PO_4_^3−^ bonds^[^ ^42^, ^43^ ^]^ from a phosphate species with unknown composition (XPO_4_). These signals are weak in the averaged spectra but show strong intensities in some areas. The phosphate species likely originate from the dispersant (BYK 180) used in the tape casting process, which contains phosphorus according to the manufacturer's data sheet.

Composite cathodes produced by co‐sintering of pre‐formed LLZO and LCO powders generally contain some amount of secondary phases formed during high temperature sintering. The most common process is ion interdiffusion at the interface between the LLZO and LCO phases (e.g., diffusion of Co into LLZO with formation of a Co:LLZO phase with simultaneous diffusion of Al from Al:LLZO, leading to Al:LCO)^[^ ^17^, ^20^, ^21^ ^]^ or even the formation of secondary phases such as Li_0.5_La_2_Co_0.5_O_4_^[^ ^17^, ^44^ ^]^ or LaCoO_3_.^[^ ^14^ ^]^ The interdiffusion products are often below the detection limit of XRD and EDS, but are accessible by Raman spectroscopy.^[^ ^17^ ^]^ The averaged Raman spectrum of the whole measured area shows the typical signals of Ta‐substituted cubic LLZO (space group *Ia*‐3*d*, highlighted in red) and rhombohedral LCO (space group $\;R\bar{3}m$, highlighted in blue)^[^ ^41^, ^45^, ^46^, ^47^, ^48^ ^]^ (Figure 4e). The LCO signals are at 488 (*E*_g_) and 596 cm^−1^ (*T*_2g_), which is very close to the spectrum of the stoichiometric LCO reference (597 cm^−1^).^[^ ^48^, ^49^ ^]^ A shift of the *T*_2g_ signal to higher wavenumbers characteristic of Li‐depleted LCO^[^ ^50^ ^]^ or a shift to higher wavenumbers indicative of Al diffusion to LCO with formation of a LiAl*_x_* Co_1−_ *_x_* O_2_ phase^[^ ^48^, ^49^, ^51^ ^]^ was not detected, therefore it can be assumed that none of these processes took place. Similarly, only the Raman signal corresponding to a stoichiometric LLZO phase was detected for the cathodes formed in situ. In the case of Co diffusion to LLZO, a strong photoluminescence signal of Co:LLZO at 693 cm^−1^ is observed,^[^ ^15^, ^17^, ^25^ ^]^ which is not the case in this work. Other possible secondary phases such as Li_0.5_La_2_Co_0.5_O_4_ at 685 cm,^−1 [^ ^17^ ^]^ La_2_CoO_4_,^[^ ^9^ ^]^ LaCoO_3_,^[^ ^14^ ^]^ La_2_O_3_,^[^ ^52^ ^]^ or La_2_Zr_2_O_7_^[^ ^14^, ^52^ ^]^ were also not detected. The Raman spectroscopy analysis therefore proves, in agreement with the EDS results, that no or only very few side reactions and ion interdiffusion occur during in situ formation of LLZO in the composite cathode. This is a clear advantage of this synthesis route, as the phases formed in these processes worsen the performance of the cathodes in full cells.

**Figure 5:**
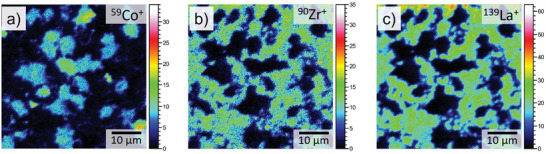
Time‐of‐flight secondary ion mass spectrometry (ToF‐SIMS) ion images for Co, Zr and La of the composite cathode tape (LLZO:LCO is 2:1).

The distribution of LCO and LLZO obtained by mapping the individual phases in the Raman spectroscopy (Figure 4f–h and the corresponding microscope image in Figure S5, Supporting Information) agrees well with the element distribution in EDS, further confirming the high phase purity of the composite cathode tapes. The same conclusion can also be drawn from the results of ToF‐SIMS measurements. ToF‐SIMS ion images (**Figure** **5**) of the same region analyzed by XRD, SEM, and Raman spectroscopy clearly show that the regions consisting of LLZO particles do not contain Co atoms. This result agrees with the results of Raman spectroscopy, according to which no Co‐ion diffusion in the LLZO grains takes place. Cobalt is mainly found inside the LCO (Figure 5a). La and Zr are homogeneously distributed in the corresponding LLZO phase (Figure 5b,c).

The combined results of the different characterization methods indicate a high phase purity of the LCO‐LLZO composite formed in situ from LLZO precursors, which positively distinguishes them from the cathodes obtained by co‐sintering of preformed LLZO and LCO powders. A small amount of impurities (below the resolution limit of XRD) most likely results from the imperfect distribution of the pre‐LLZO powders during slurry production and can be minimized by optimizing the milling and stirring steps. The XRD analysis of the tapes sintered at different temperatures shows that the formation of LLZO from metal oxide precursors requires temperatures higher than 750 °C (the temperature of 1050 °C was used in this work). At temperatures of 650 °C (corresponding to the temperature of the debinding step), the XRD analysis shows the presence of metal oxide precursors and the LCO phase in the cathode tape (Figure 3). In addition, two new phases are formed, which can be assigned to La_2_Zr_2_O_7_ and Li_0.5_La_2_Al_0.5_O_4_. These phases are also present in the tape calcined at 750 °C, but the formation of some cubic LLZO phase is also visible. These results are in good agreement with literature reports, as La_2_Zr_2_O_7_ was found to be the most common intermediate phase at lower temperatures during LLZO synthesis. For example, Rao et al.^[^ ^53^ ^]^ showed that during the synthesis of LLZO:Ta, the initial glass‐ceramic precursor mixture fully crystallizes at temperatures of up to 540 °C to form intermediate products such as La_2_O_2_CO_3_, La_2_Zr_2_O_7_ and some initial precursors. Li_0.5_La_2_Al_0.5_O_4_ has been found for Al‐doped LLZO synthesized in a sol–gel reaction.^[^ ^54^ ^]^ Chen et al. showed the formation of Li_2_ZrO_3_, La_2_Zr_2_O_7_, and Li_0.5_La_2_Al_0.5_O_4_ for Al:LLZO with similar precursors as in this study. A maximum amount of La_2_Zr_2_O_7_ and Li_0.5_La_2_Al_0.5_O_4_ phases were found at temperatures between 750 and 800 °C, while the amount of La_2_O_3_ and its carbonates and hydroxides was decreasing at these temperatures.^[^ ^55^ ^]^ This could explain that in this study only La_2_Zr_2_O_7_ and Li_0.5_La_2_Al_0.5_O_4_ are detected during heat treatment, as all precursors and other phases formed are already decomposed at 650 °C. In addition, no Li_2_ZrO_3_ is detected in the tapes sintered at 650 and 750 °C, although some should be visible at 650 °C.^[^ ^53^, ^56^ ^]^ We assume that at 650 °C the precursors are already reacted to the intermediate products Li_0.5_La_2_Al_0.5_O_4_ and La_2_Zr_2_O_7_ doped with Ta. The remaining Li is then available for the formation of LLZO around the melting point of Li_2_CO_3_ or the decomposition of LiOH·H_2_O at 713 °C.^[^ ^56^, ^57^ ^]^

### 2.2 Electrochemical Performance of LCO‐LLZO Composite Cathode Tapes

The electrochemical performance of the composite cathode tapes was investigated in conventional liquid electrolyte based cells and in a solid‐state model cell specifically designed for the incorporation of free‐standing cathodes without additional temperature treatment steps, as described in our previous work.^[^ ^28^ ^]^ A composite cathode tape with a thickness of 70 µm and an active material loading of 13.8 mg was assembled in a CR2032 coin cell for conventional cell tests. For the solid‐state cell setup, the cathode tape with a thickness of 85 µm and an LCO loading of 15 mg (2.1 mAh cm^−1^) was glued onto a dense LLZO:Ta separator connected to a Li metal foil anode by an ion‐conducting PEO polymer interlayer to (cell configuration: Li|LLZO|PEO|LCO‐LLZO|Au). The measurements in liquid electrolyte were performed at 25 °C, while the solid‐state cell was characterized at 60 °C due to the poor ionic conductivity of PEO at room temperature (5.4 × 10^−5^ S cm^−1^ at 30 °C).^[^ ^23^, ^58^ ^]^

**Figure 6:**
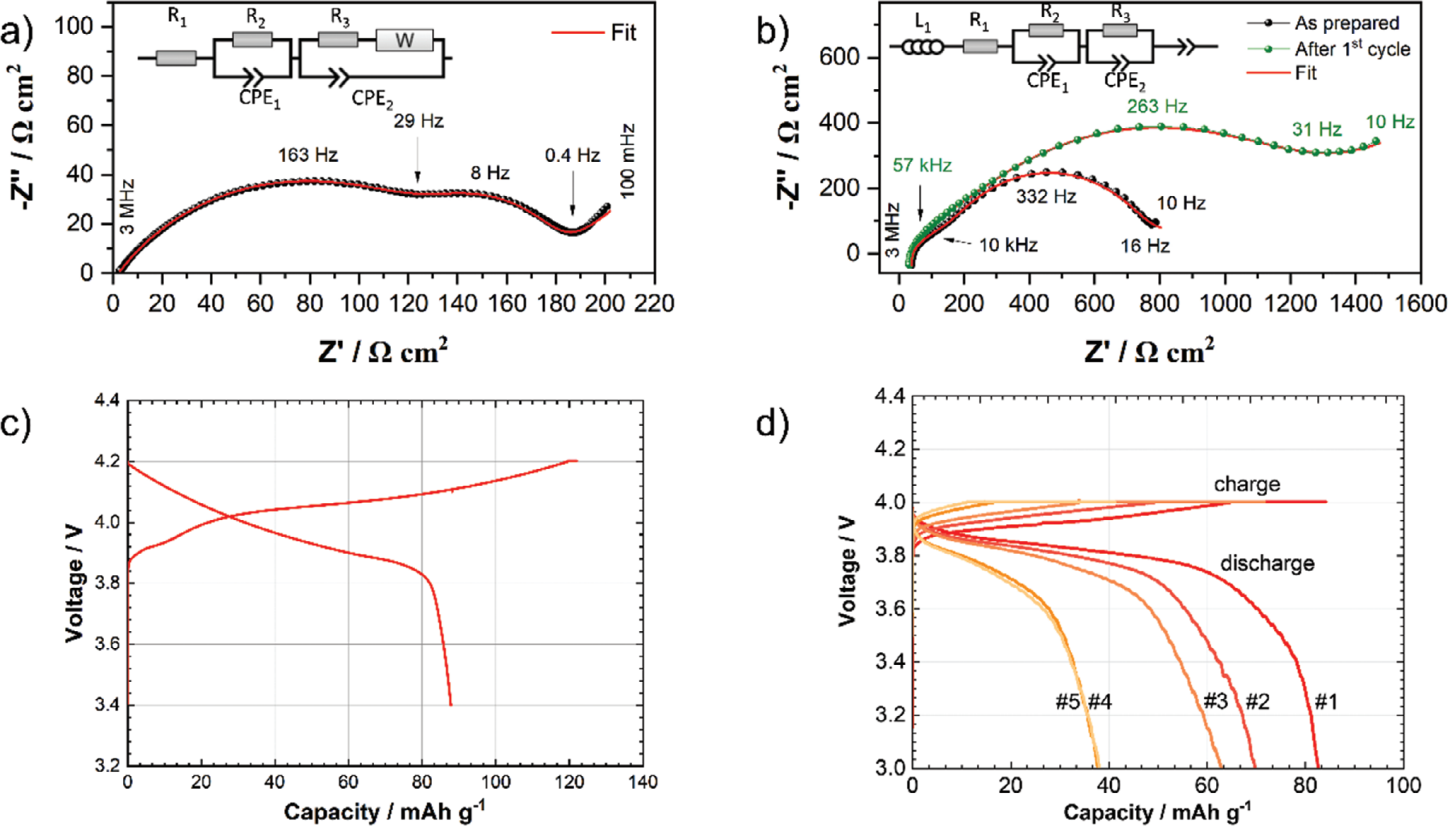
Electrochemical characterization of a Nyquist plot of the electrochemical impedance spectrum a) in a conventional liquid electrolyte‐based cell at 25 °C, after the first charging step and b) in a solid‐state cell at 60 °C in the as prepared state as well as after the first cycle. The equivalent circuit models used for fitting the data are depicted. Galvanostatic charge–discharge profiles c) in a conventional liquid electrolyte‐based cell at 30 µA cm^−2^ and d) in a solid‐state cell at 35 µA cm^−2^ for five cycles for an ASB‐cell.

The Nyquist plots of the electrochemical impedance spectrum for the conventional liquid electrolyte‐based cell (**Figure** **6**a) show two semicircles and can be fitted with the equivalent circuit shown in Figure 6a. The serial ohmic resistance (2 Ω cm^2^) originates from all bulk materials (electrolyte, separator, current collectors, etc.). The broad suppressed high frequency semicircle corresponds to the most resistive solid interfaces within the composite cathode tape. The value of 145 Ω cm^2^ (10^−7^ F) is in the same range as the impedance of LCO‐LLZO interface reported by us previously for various solid LCO‐LLZO cathodes.^[^ ^16^ ^]^ The second semicircle at mid‐ to low‐ frequencies originates from the charge transfer resistance (34 Ω cm^2^, 10^−3^ F). The straight line in the low frequency range of the Nyquist plot is represented by the Warburg impedance and originates from the Li‐ion diffusion in the cathode material. All values can be found in Figure S7 (Supporting Information).

The Nyquist plots of the electrochemical impedance spectrum for the solid‐state cell (Figure 6b) show higher ohmic resistance (≈30 Ω cm^2^ for both spectra shown in Figure 6b) due to a higher resistance of LLZO separator and PEO connecting layer in the used cell setup. For the as prepared as well as for the cycled solid‐state cell (Figure 6b), the capacitances (10^−8^ F) of the high frequency semicircles fit to the values typically reported for grain boundaries of LLZO.^[^ ^59^ ^]^ The corresponding resistances of 97 and 123 Ω cm^2^ for the as prepared and cycled cell, respectively, can be therefore assigned to the LLZO grain boundaries in the separator and in the composite cathode. Similar to the impedance spectrum of the liquid cell discussed above, the Nyquist plot for the solid‐state cell shows an additional semicircle in the high‐ to medium‐frequency range that can be attributed to a most resistive interface in the composite cathode, which is dominated by LCO‐LLZO interface contribution. For the as prepared state, fitting leads to an interfacial LCO‐LLZO resistance of 544 Ω cm^2^ (10^−7^ F), which nearly doubles after the first charge/discharge cycle to 1354 Ω indicating a severe interfacial degradation during cycling (Figure 6d).

The impedance of LCO‐LLZO interface in the in situ prepared cathodes and the total cell resistance are  lower than the values we previously reported for composite LCO‐LLZO cathodes prepared from pre‐synthesized LLZO particles.^[^ ^15^, ^16^, ^19^, ^26^, ^28^ ^]^ One reason for the lower interfac resistance could be much lower cation interdiffusion between the LCO and an in situ formed LLZO, especially the suppressed diffusion of Co into the LLZO lattice as described above. A much cleaner and more defined interface in the in situ‐formed cathode may also explain the remarkably low total resistance of about 0.6 kΩ cm^2^ for the as‐assembled solid‐state cell, which is much lower than the total cell resistance of 10 kΩ cm^2^ obtained by us previously for the similar cell setup containing LCO‐LLZO cathode tapes with similar morphology prepared from pre‐synthesized LLZO powders.^[^ ^25^, ^28^ ^]^

The liquid electrolyte‐based cell was cycled between 3.4 and 4.2 V at a constant current density of 30 µA cm^−2^ (0.01 C, Figure 6c). An initial discharge capacity of 88 mAh g^−1^ was obtained, which corresponds to 63% of the theoretical capacity of LCO (140 mAh g^−1^). These results show that LCO remains active during tape preparation and LLZO crystallization, although the reduced capacity indicates that its accessibility is limited under the chosen conditions of electrochemical measurements. One of the main reasons for the reduced capacity utilization is a low rate of coupled electron/ion transport in thick ceramic composite cathodes at room temperature, as shown by Danner et al.,^[^ ^40^ ^]^ since no conductive additive was added to the tape and the electronic conductivity is provided solely by the LCO phase. The composite cathodes fabricated in this work have a large thickness of 70–85 µm (and thus a high loading of cathode active material), which exceeds the theoretical limit of 50 µm at which the transport limitations calculated for this type of cathode morphology can be avoided.

In general, the capacity utilization of ceramic cathodes in liquid electrolytes is higher than in the solid‐state, since the liquid electrolyte can penetrate into the pores and thus increase the ionic conductivity of the composite cathodes. Surprisingly, the in situ formed cathode tapes show virtually the same capacity utilization in the liquid and in the solid‐state cells, with initial discharge capacity of 82 mAh g^−1^ at 60 °C for 85 µm thick tapes, which is 59% of the theoretical capacity of LCO (Figure 6d). Similar performance of the in situ formed cathodes in liquid and solid‐state cells can be attributed to a very high relative density of the tapes and a closed porosity that makes them practically impermeable to the liquid electrolyte, which is in a good agreement with the µCT tomography results discussed above.

Like all previously reported all‐ceramic LCO‐LLZO composite cathodes, the in situ formed LCO‐LLZO cathodes show a strong decrease in capacity already after the second cycle, reaching half of the initial capacity after the fifth cycle. FIB‐SEM examination of the cycled cathode tapes shows their significant mechanical degradation with formation of a large number of microcracks, already after the first charging cycle (Figure S8, Supporting Information) due to the volume change during charging and discharging and an irreversible loss of contact between the LLZO and LCO particles as the main reason for the capacity drop.^[^ ^39^, ^60^ ^]^

The performance of in situ formed composite electrodes is comparable to, but slightly worse than, that of LCO‐LLZO cathodes fabricated from preformed particles for which the first discharge capacity of 100 mAh g^−1^ and above (70% of theoretical capacity) is typically achieved.^[^ ^15^, ^25^ ^]^ We assume that the capacity utilization and cycling stability of the cathodes formed in situ can be significantly increased by further optimization of the manufacturing process. In particular, optimization of the composition of the LLZO phase, which can be easily achieved by choosing the type and stoichiometry of the precursor compounds (including the dopants^[^ ^40^ ^]^); optimization of the milling step and composition of the slurry; further optimization of the casting sequence to improve the gradient structure and reduce the tape thickness, and optimization of the thermal treatment should be performed to increase the electrochemical performance of the in situ formed ceramic cathodes. Although the initial capacity of the composite cathodes can be greatly improved by optimizing the fabrication and sintering techniques and up to 100% of the theoretical capacity can be achieved in the best reported systems, the low cycling stability with a continuous capacity drop remains one of the most persistent problems of LLZO‐based ceramic composite cathodes that has not yet been solved.^[^ ^25^, ^44^, ^61^ ^]^ The recent publications on the engineering design of LCO‐LLZO interface and the development of protective coatings that mitigate the degradation of the interface pave the way for further performance improvement and may be used to improve the cycling stability of in situ formed ceramic cathodes in the future.^[^ ^19^ ^]^

## 3 Conclusion

For the first time, a method has been found to integrate the complex synthesis of garnet‐based LLZO directly into the production of oxide ceramic‐based mixed cathodes for solid‐state batteries. By completely eliminating the conventional synthesis steps of LLZO, which usually take several days and require multiple heating steps, the developed process minimized energy and time consumption. Surprisingly, the common problem of cobalt ion diffusion in LLZO during the sintering of calcined powders is completely suppressed in this new approach.

Furthermore, it has been shown to be suitable even for the fabrication of design‐optimized composite cathodes that exhibit a gradient microstructure to enhance performance. While the overall performance still needs to be further optimized, the manufactured full cells already show a quite low overall resistance compared to similar cell designs in the literature due to the high density of the cathode material. The new process thus represents an economical, environmentally friendly, scalable, one‐step manufacturing approach for LLZO‐LCO composite cathodes, moving them further toward economic feasibility for industrial applications.

## 4 Experimental Section

### Composite Cathode Fabrication

The precursor powders LiOH·H_2_O (Merck, 98%), Al_2_O_3_ (Inframat, 99.9%), La_2_O_3_ (Merck, 99% pre‐dried at 900 °C for 10 h), ZrO_2_ (Treibacher, 99.7%), Ta_2_O_5_ (Inframat, 99.95%) with 10% Li excess to compensate for lithium evaporation during high temperature treatment were weighed in according to stoichiometry Li_6.54_Al_0.02_La_3_Zr_1.6_Ta_0.4_O_12_ (LLZO:Ta). The powder was wet milled in ethanol (97%, Hoffmann–Schmittmann GmbH) in a high energy ball mill (E_MAX_, Retsch) at 1000 rpm for 15 min with zirconia balls of 1 mm diameter. The LCO powder (MSE Supplies, 99.5%) was wet milled separately in ethanol in a planetary ball mill (Pulverisette P7, Fritsch) at 500 rpm for 240 min with zirconia oxide balls of 3 mm diameter. For the LLZO precursor mixture, the solvent and moisture removal were performed in a drying step in an N_2_ atmosphere at 60 °C for 12 h. The LCO milling suspension was dried on a hot plate in the air at 70 °C for 12 h.

The received LLZO precursor and LCO powder were mixed with organic additives and zirconia balls (3 mm) to decrease agglomerates in a planetary mixer (Thinky) at 1500 rpm for 2 min. The compounds and stoichiometry of the organic matrix can be found in a previous publication containing, for example, plasticizers such as BYK180.^[^ ^62^ ^]^ The final tape‐cast slurries contain different LLZO:LCO powder ratios, namely 2:1, 1:1, and 1:2, with around 13 wt% organic. All three slurries were sequentially tape‐cast with a total gap bar height of 240 µm. Afterward, the tape was dried for 15 h in air and then hot pressed (16 kN cm^−2^, 80 °C, P/O/Weber GmbH). After hot pressing, the material was punched into disks of 12 mm diameter, and the green tapes were sintered in an alumina crucible on a MgO sheet and LLZO + LCO sacrificial powder (LLZO synthesized by solid‐state reaction^[^ ^62^ ^]^ and LCO (MSE Supplies)). Finally, the material was sintered in air at 1050 °C for 2 h with an organic burn‐out step at 650 °C for 2 h, all with a heating ramp of 5 K min^−1^.

### Material Analysis

To obtain information about the phase purity and crystal structure of the samples, XRD measurements were performed. The sample at 1050 °C sinter temperature has been measured on a Bruker D4 Endeavour instrument using Cu‐Kα radiation and equipped with a 1D detector LYNXEY and a DIFFRACplus BASIC package, which was released in 2009, from 10° to 80° 2Θ in 0.02° steps. The Rietveld analysis was performed using the TOPAS software from Bruker.^[^ ^63^ ^]^ Samples at 650 and 750 °C were measured on an Empyrean (Malvern Panalytical, Almelo, Netherlands), using a 255‐channel PIXcel linear detector from 10° to 90° 2Θ in 0.026° steps. The morphology and microstructure were investigated by SEM (Zeiss Gemini 450) using a secondary electron (SE) and BSE detector and an EDS detector (ULTIM MAX 170, OXFORD INSTRUMENTS). The electron acceleration voltage was set to 8 kV, except for the overview of the full cathode (Figure 3a) it was set to 15 kV. Powder samples were prepared on a Si wafer, placed with pure ethanol, dried, and coated with platinum (<10 nm, Baltec, type SCD 050). The sintered tape was embedded in epoxy resin, ground by SiC paper, and polished with a water‐free diamond suspension for cross‐section analysis. After the final polishing step a thin platinum layer was sputtered on top (<10 nm, Baltec, type SCD 050). Porosity was estimated based on the digital analysis of the SEM images with ImageJ software.^[^ ^64^ ^]^

Potential Cobalt diffusion into LLZO grains and Al diffusion into LCO grains were examined by TOF‐SIMS. The measurements were performed on a TOF‐SIMS 5 NCS system (IONTOF GmbH) equipped with a Bi Nanoprobe 50 primary ion gun, an oxygen sputter gun, and a low‐energy electron flood gun for charge compensation. Bi^+^ primary ions were chosen for analysis at an energy of 30 keV, and the oxygen sputter gun operated at 1 keV. Images were taken in positive polarity in an analysis raster of 50 µm × 50 µm and 1024 × 1024 pixels in imaging mode, while the sputter raster was set to 350 µm × 350 µm.

### Raman Spectroscopy Coupled with SEM

Raman spectroscopy (invia qontor, renishaw) was measured with a 532 nm laser (≈2.5 mW) and a 2400 l mm^−1^ grating installed. The spectra were collected with a step size of 1 µm in *x*‐ and *y*‐direction and a measuring time of 1 s per spectrum. The spectra were processed, including cosmic ray removal and normalization, and the mappings were averaged to a single spectrum, or a phase analysis of the mapping was performed. For the microstructure investigation, the samples were sputtered with an Au layer and analyzed with an SEM (EVO 15, zeiss) using a BSE detector together with an EDS detector (ultim max 100, oxford instruments) with an acceleration voltage was set to 15 kV.

### Micro X‐Ray Tomography

µCT measurements were performed with a Zeiss Xradia Versa 520 to assess the electrode microstructure on a larger scale. Cathode samples were carefully cracked into smaller pieces (<10 × 10 mm) and fixed on top of a thin metal needle. The X‐ray source was set to an acceleration voltage of 60 kV and a power of 5 W while using 40x optical magnification, resulting in a voxel length of 300 nm. Image quality was ensured through high exposure time of 35 s per image, leading to an overall measurement time of roughly 30 h. An initial warmup step ensures the sample has a constant temperature during the tomography. The chosen parameter set provides high‐quality images with good contrast. This lowers the number of post‐processing steps to just one non‐local means filter, which was implemented in the 2023 distribution of GeoDict (GeoDict software, Math2Market, Germany, release 2024). Finally, Otsu's method was chosen as the segmentation algorithm.

Microstructural evaluation was also performed with GeoDict 2023, using the MatDict and PoroDict modules. Initially, the phase distribution of active material and electrolyte was analyzed using MatDict. In the second step, pore connectivity was quantified in PoroDict.

### Cell Assembly and Electrochemical Characterization

To assemble the solid‐state battery, a thin Au current collector was deposited on the LCO‐rich side of the tape‐cast composite cathode by sputtering (5–15 nm, Cressington 108auto Coater). LLZO pellets (prepared as described by Scheld et al.^[^ ^17^ ^]^) with a diameter of 11.4 mm and a thickness of 760 µm were used as separators. The pellets were polished with SiC paper (4000 grit) to remove surface impurities and to ensure good contact with the Li metal anodes. The Li metal disks used as anodes were punched from freshly calendered lithium foil, manually applied to the LLZO pellet with light pressure and heated to 300 °C for 5 min to melt Li and improve contact. The Li metal anodes had a diameter of 11 mm and a thickness of about 125 µm. The composite cathode tape and the LLZO separator with Li anode were bonded with a polymer layer, prepared from a solution of PEO (*M*_v_ 600 000, Sigma‐Aldrich), LiTFSI (Sigma‐Aldrich), and conductive carbon (Alfa Aeser) in acetonitrile in an Ar‐filled glovebox. The ratio of PEO to LiTFSI was 1:20. The polymer solution was applied to the side of the cathode tape that was not coated with Au and attached to the LLZO separator. The assembled cell was dried for 5 h at room temperature in an argon atmosphere so that the polymer layer had a thickness of 35 µm after drying. The electrochemical measurements were carried out in a Swagelok‐type cell. The full cells were equilibrated at 60 °C for 6 h prior to electrochemical characterization to ensure good contact between the PEO layer and the ceramic components.

Electrochemical tests were carried out at 60 °C by using a VMP‐300 multi‐potentiostat (BioLogic Sciences Instruments Ltd, France) combined with a climate chamber (Vötsch Industrietechnik VT 4002EMC, Germany). The data were evaluated using the EC‐lab software (BioLogic Sciences Instruments Ltd, France). Galvanostatic cycling was carried out at 35 µA cm^−2^ in a voltage window of 3.0–4.0 V versus Li/Li^+^. The battery was charged to a constant voltage of 4.0 V versus Li/Li^+^ until the current dropped to 13 µA cm^−2^. Electrochemical impedance spectroscopy (EIS) was performed at 3.0 V in a frequency range of 3 MHz to 10 Hz with an amplitude of 10 mV.

Further electrochemical investigations using a liquid electrolyte were carried out in coin cells (CR2032). The cells were assembled in an argon‐filled glovebox by using the tape‐cast composite cathode (LCO‐LLZO), a glass fiber separator (Whatman), and a Li‐metal foil (Alfa Aesar) as an anode. A 1.0 m solution of LiPF_6_ (Sigma‐Aldrich) in ethylene carbonate (EC)/dimethyl carbonate (1:1 by volume) was used as electrolyte. Electrochemical tests with liquid electrolytes were conducted at 25 °C using the previously mentioned VMP‐300 multi‐potentiostat and climate chamber. The data were evaluated using the software EC‐lab (BioLogic Sciences Instruments Ltd, France) and Data Analysis (Batalyse GmbH, Germany). Galvanostatic cycling was carried out at a current density of 31 µA cm^−2^ in a voltage window of 3.4–4.2 V versus Li/Li^+^. The battery was charged to a constant voltage of 4.2 V versus Li/Li^+^ until the current dropped to 15 µA cm^−2^. EIS was performed at 4.2 V in a frequency range of 3 MHz to 100 mHz with an amplitude of 10 mV. The software RelaxIS 3 (rhd instruments GmbH and Co. KG, Germany) was used to evaluate all impedance spectra.

## Conflict of Interest

The authors declare no conflict of interest.

## Author Contributions

V.K. conceived and conducted the experiments. C.S. performed ToF‐SIMS measurements. W.S.S. performed Raman and EDS measurements. A.L. performed µCT measurements. C.R. performed electrochemical characterization in liquid electrolyte‐based cells. V.K., C.S., and W.S.S. prepared the original draft. V.K., A.L., C.R., W.M., M.F., D.F., and O.G. reviewed and edited the manuscript. Funding acquisition and project administration were conducted by M.F., D.F., and O.G. The concept was initially designed by V.K., M.F., and O.G.

## Supporting information

Supporting Information

## Data Availability

The data that support the findings of this study are available from the corresponding author upon reasonable request.
