# Supplementary material for: Direct Precursor Route for the Fabrication of LLZO Composite Cathodes for Solid‐State Batteries
Source: Adv Sci (Weinh). 2024 Sep 19;11(42):2404682. doi: 10.1002/advs.202404682 (PMC11558113; doi:10.1002/advs.202404682)
Supplement: Supplementary file 1 — Supporting Information [file ADVS-11-2404682-s001.docx]

Supporting Information

Direct precursor route for the fabrication of LLZO composite cathodes for solid-state batteries

*Vivien Kiyek, Christian Schwab, Walter Sebastian Scheld, Christoph Roitzheim, Adrian Lindner, Wolfgang Menesklou, Martin Finsterbusch, Dina Fattakhova-Rohlfing*, Olivier Guillon*


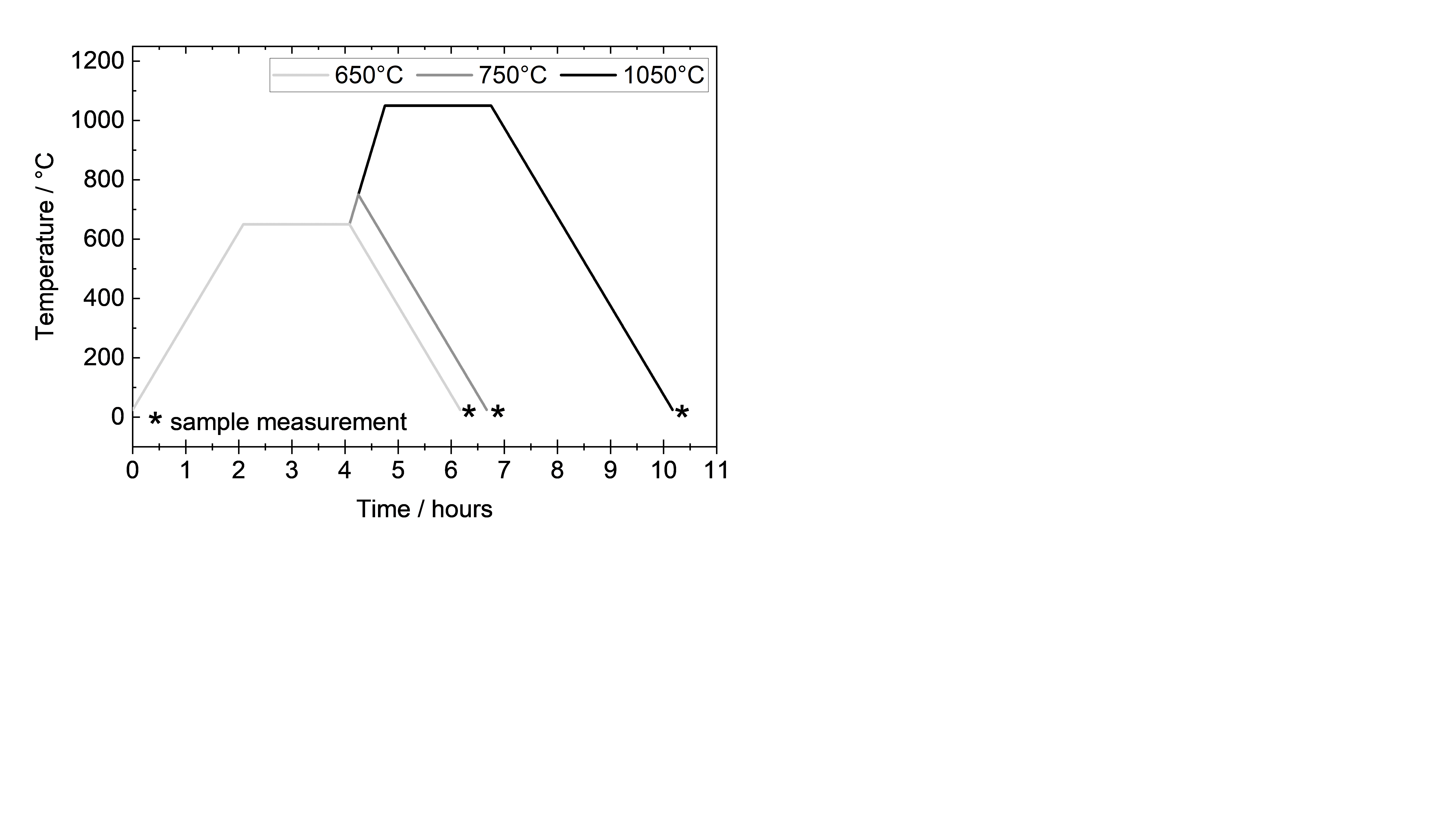


**Figure S1:** Heat profile of sinter treatment and indication of the points, where XRD samples were extracted for analysis.


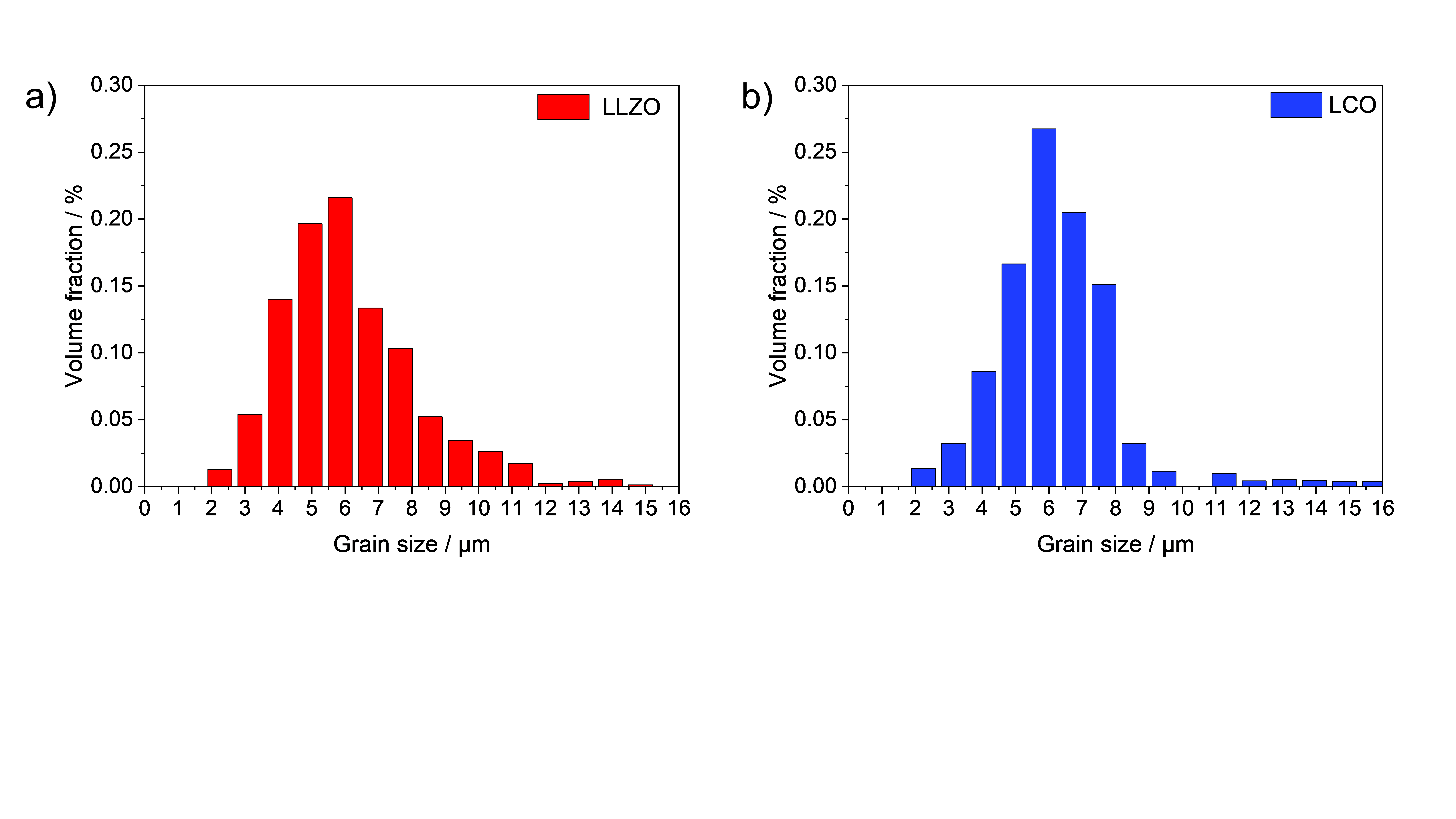


**Figure S 2:** Grain size distribution of a) LLZO and b) LCO, taken from µCT measurements.


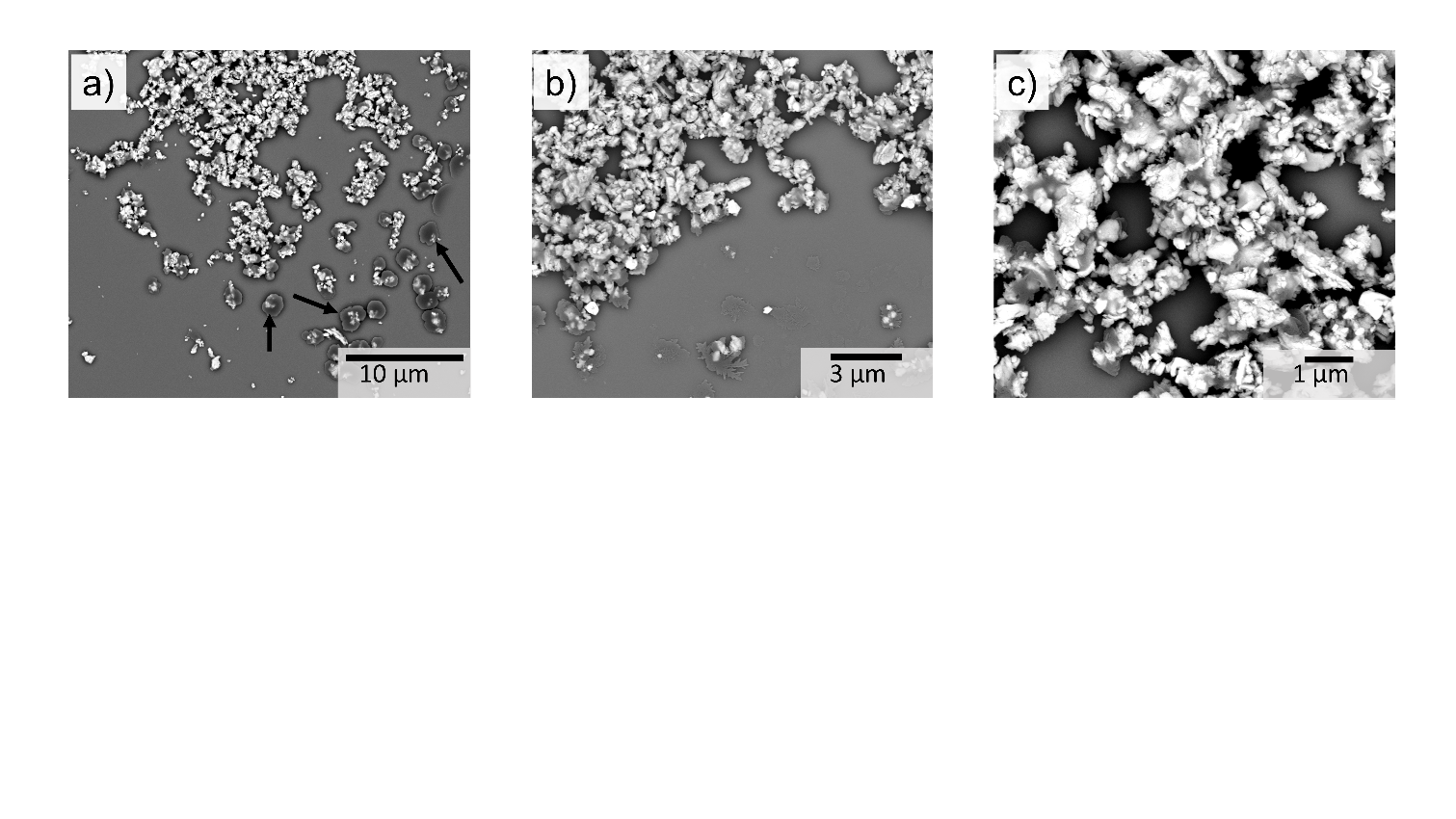


**Figure S3:** BSE-SEM of precursor particles after milling on Si wafer in three different magnifications.


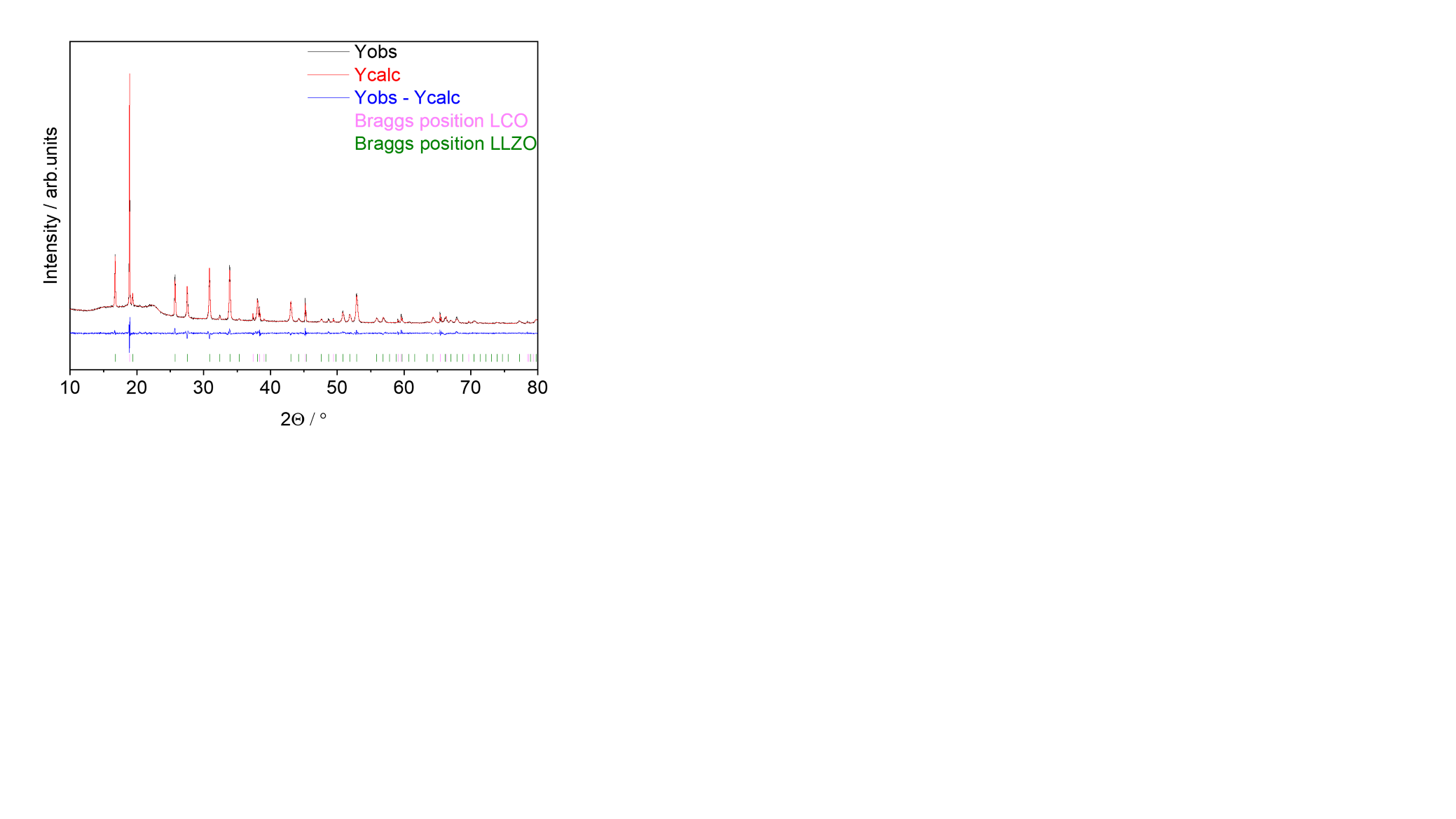


**Figure S4:** Rietveld refinement of the XRD of sintered composite cathode (at 1050 °C) with LLZO:Ta and LCO as Braggs position.


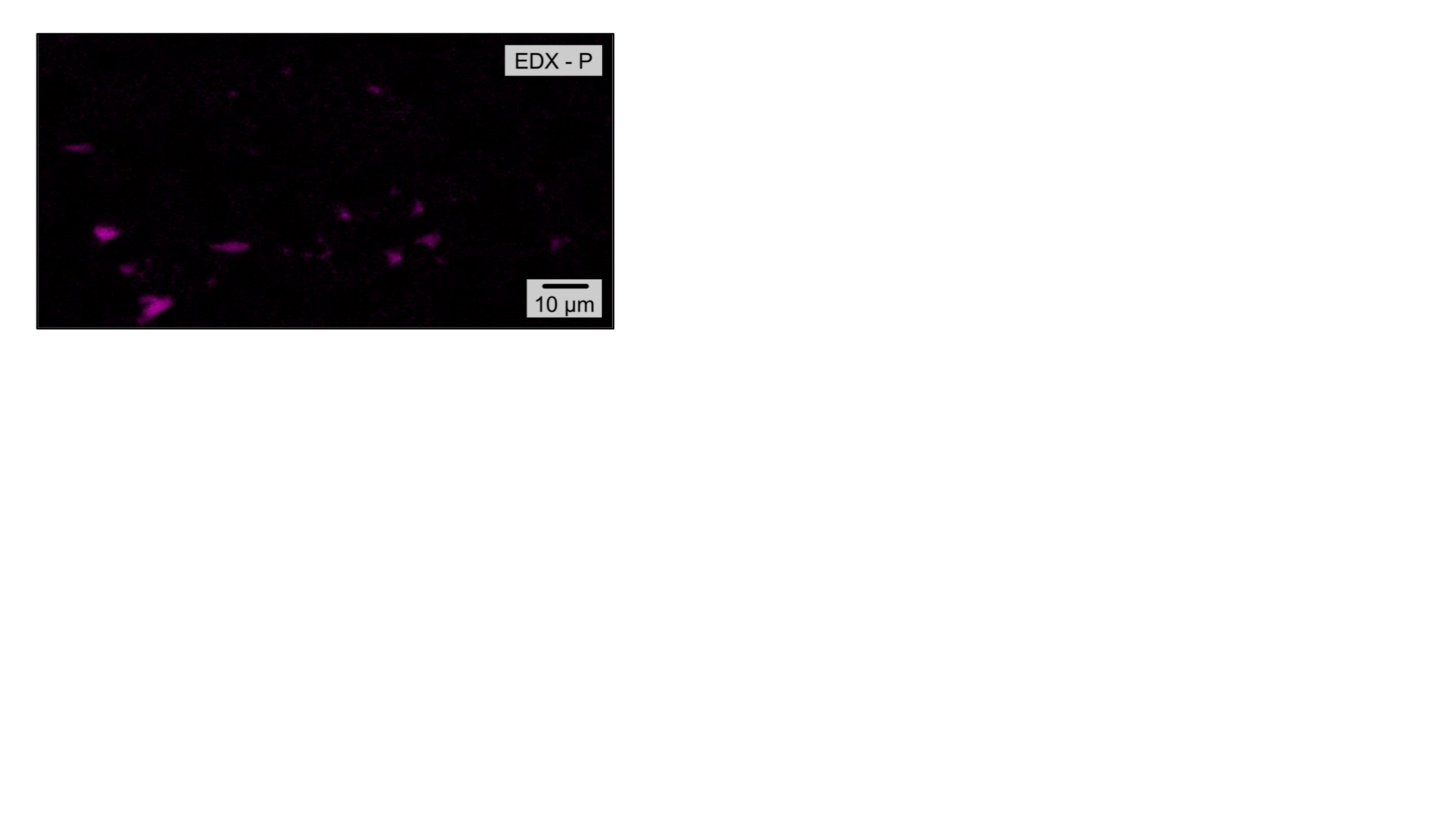


**Figure S5:** EDS P intensity mapping


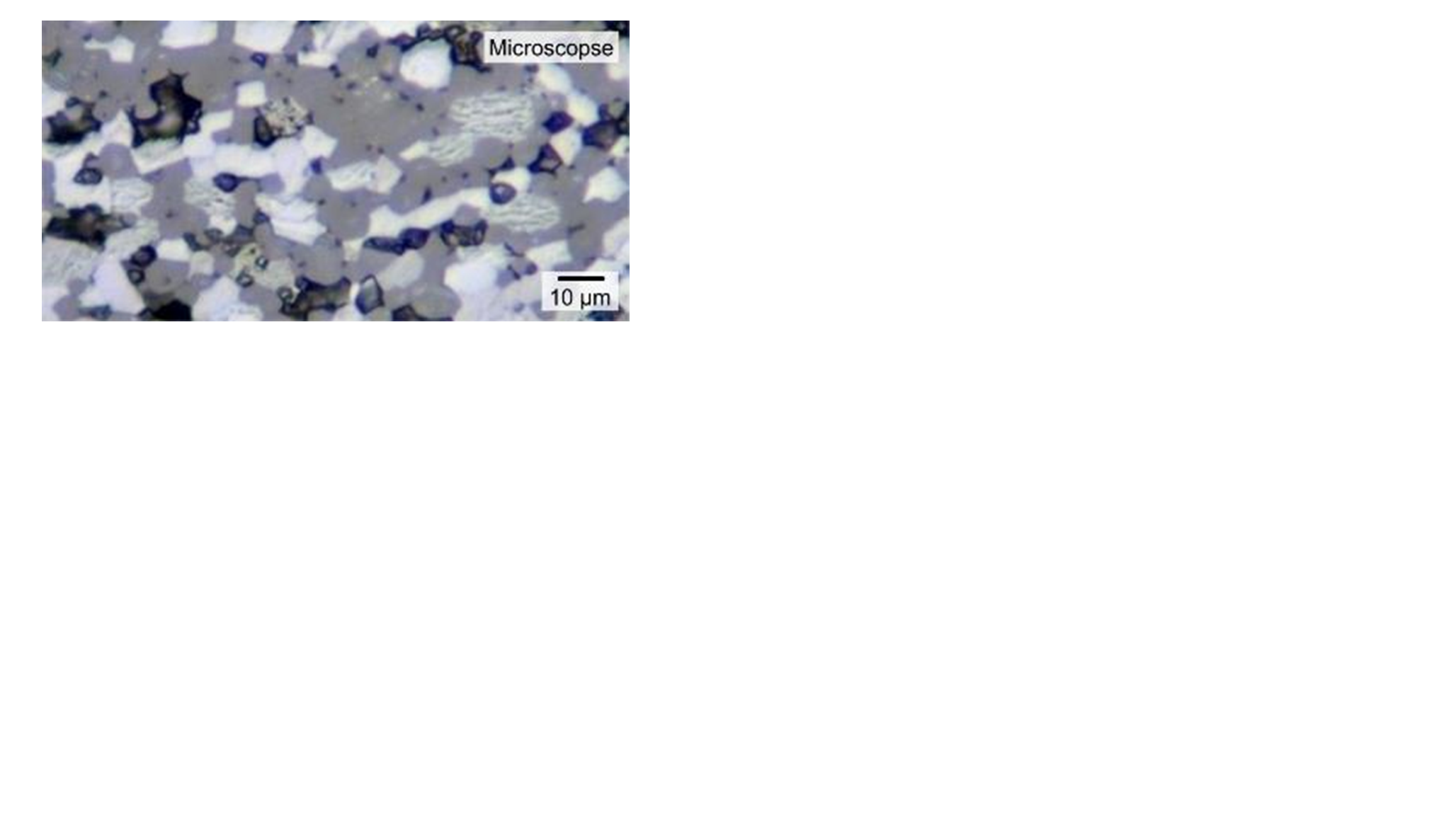


**Figure S6:** Microscope image of the same region like in the SEM image in Figure 4a


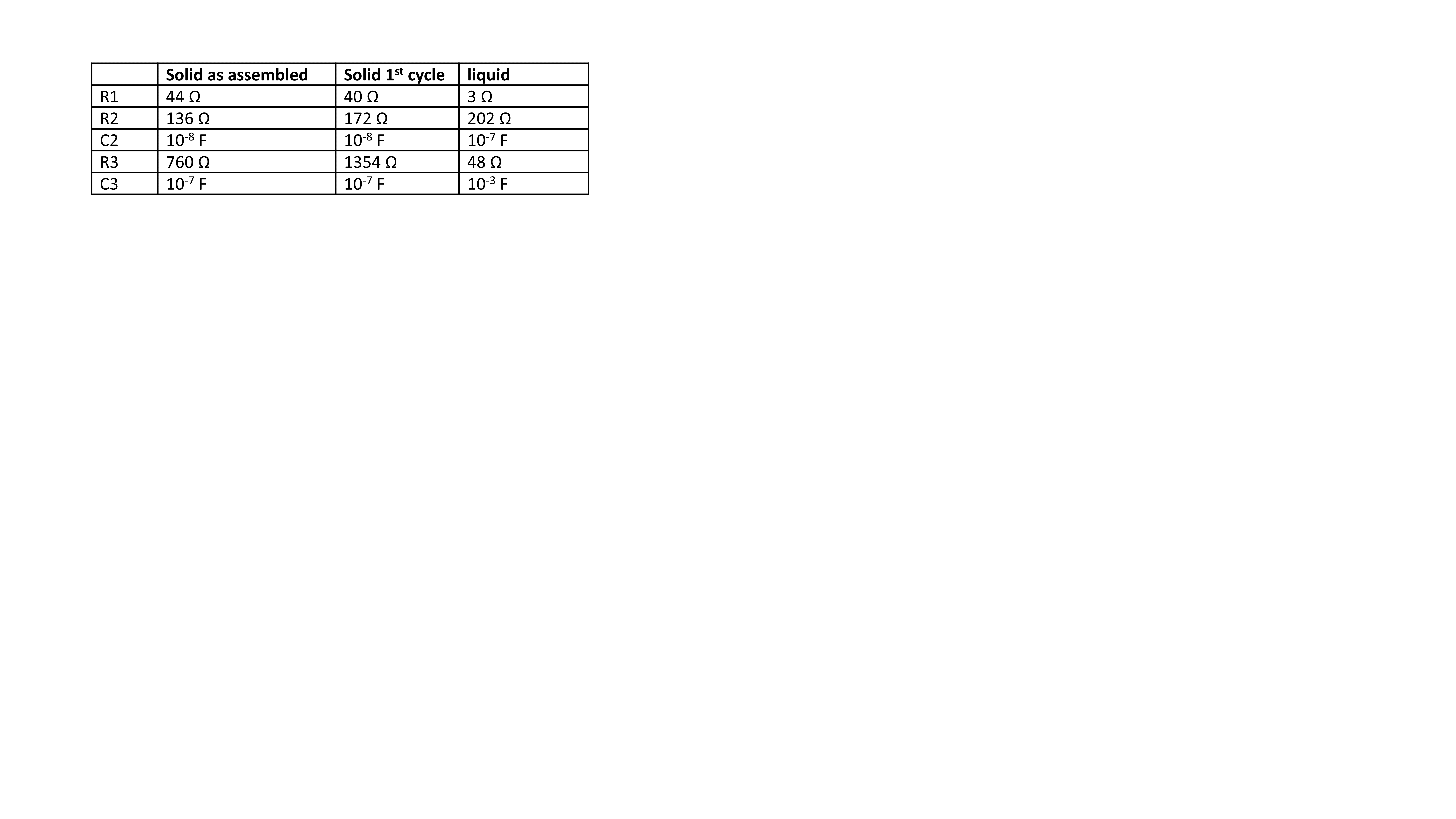


**Figure S 7:** Values of the impedance fitting data for the solid-state cell as prepared, after its first cycle and for the liquid electrolyte based cell.


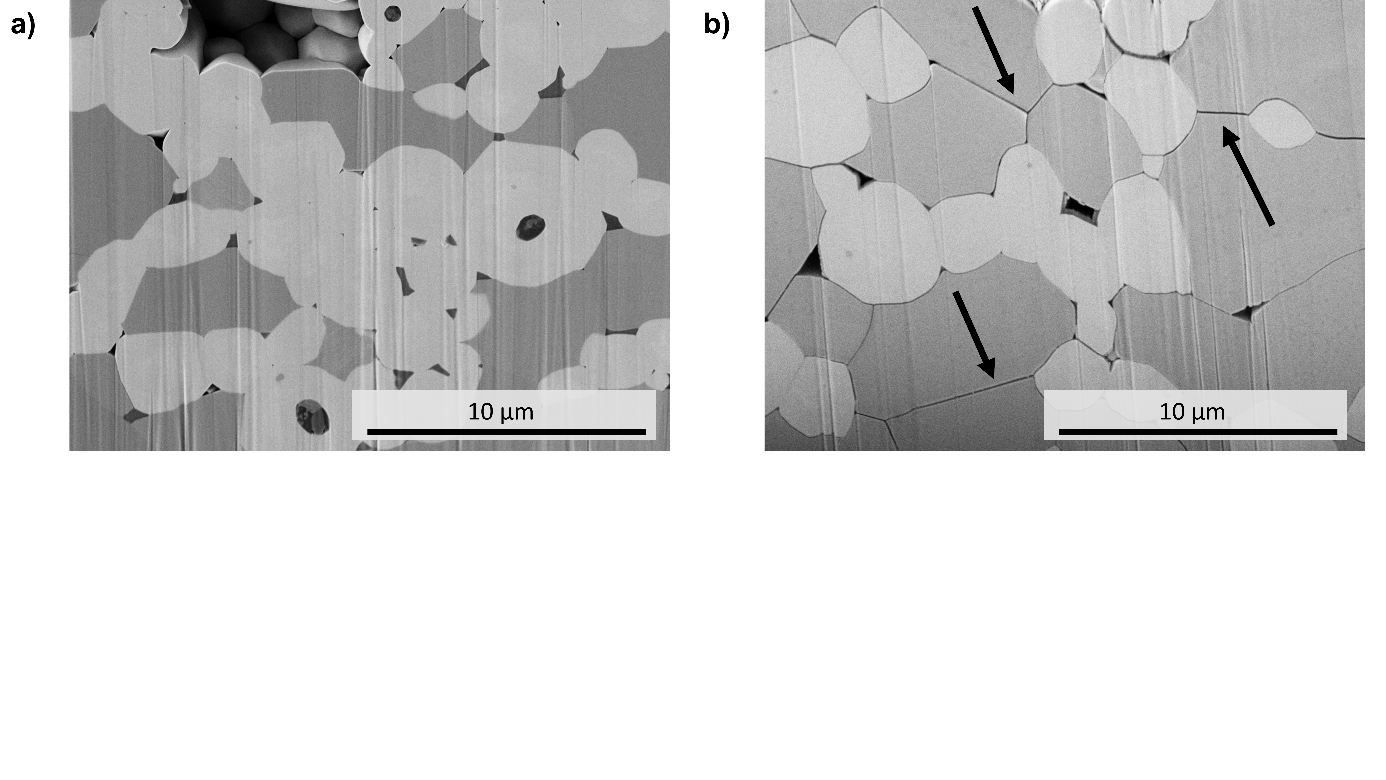


**Figure S 8:** FIB-SEM micrographs were obtained using a Zeiss 1540 XB equipped with a Ga-ion source. Cross sections were milled using a beam current of 2 nA. To prevent any charge-up artifacts, thin platinum layers were deposited on top of the samples. a) shows a composite cathode before cycling, while b) shows the cell after a cycle, with crack formation indicated by arrows.
